# Is AI useful in psychotherapeutic education?

**DOI:** 10.1192/j.eurpsy.2026.10306

**Published:** 2026-07-31

**Authors:** F. H. Kraxner

**Affiliations:** Forensic psychiatry, Psychiatric Services of Kanton Aargau (PDAG), Brugg/Windisch, Switzerland

## Abstract

**Abstract:**

Artificial intelligence (AI) is increasingly entering mental health care and education, raising important questions about its role in training the next generation of psychiatrists. This talk explores whether and how AI can meaningfully contribute to psychotherapeutic education for early career psychiatrists, while maintaining core human values such as empathy, clinical judgment, and therapeutic alliance.

Drawing on current developments and practical examples, the presentation discusses potential benefits of AI-assisted learning, including personalized training, simulated clinical scenarios, feedback on therapeutic skills, and improved access to educational resources across global contexts. At the same time, ethical considerations, limitations, and risks—such as over-reliance on technology, bias, and impacts on professional identity—are critically examined.

By situating AI within the broader goals of improving quality of care and expanding horizons in mental health education, this talk aims to provide a balanced perspective on how AI can support, but not replace, human-centered psychotherapy training.

**Disclosure of Interest:**

None Declared

